# Concordance between whole‐exome sequencing and clinical Sanger sequencing: implications for patient care

**DOI:** 10.1002/mgg3.223

**Published:** 2016-05-10

**Authors:** Alison Hamilton, Martine Tétreault, David A. Dyment, Ruobing Zou, Kristin Kernohan, Michael T. Geraghty, FORGE Canada Consortium, Care4Rare Canada Consortium, Taila Hartley, Kym M. Boycott

**Affiliations:** ^1^ Children's Hospital of Eastern Ontario Research InstituteUniversity of OttawaOttawaOntarioCanada; ^2^ Department of Human Genetics McGill UniversityMontréalQuébecCanada; ^3^ McGill University and Genome Québec Innovation CenterMontréalQuébecCanada; ^4^ Department of GeneticsChildren's Hospital of Eastern Ontario ResearchOttawaOntarioCanada; ^5^ Division of Metabolics and Newborn ScreeningDepartment of PediatricsChildren's Hospital of Eastern OntarioOttawaOntarioCanada

**Keywords:** Coverage, rare diseases, Sanger sequencing, whole‐exome sequencing

## Abstract

The clinical translation of next‐generation sequencing has created a paradigm shift in the diagnostic assessment of individuals with suspected rare genetic diseases. Whole‐exome sequencing (WES) simultaneously examines the majority of the coding portion of the genome and is rapidly becoming accepted as an efficient alternative to clinical Sanger sequencing for diagnosing genetically heterogeneous disorders. Among reports of the clinical and diagnostic utility of WES, few studies to date have directly compared its concordance to Sanger sequencing, which is considered the clinical “gold standard”. We performed a direct comparison of 391 coding and noncoding polymorphisms and variants of unknown significance identified by clinical Sanger sequencing to the WES results of 26 patients. Of the 150 well‐covered coding variants identified by Sanger sequencing, 146 (97.3%) were also reported by WES. Nine genes were excluded from the comparison due to consistently low coverage in WES, which might be attributed to the use of older exome capture kits. We performed confirmatory Sanger sequencing of discordant variants; including five variants with discordant bases and four with discordant zygosity. Confirmatory Sanger sequencing supported the original Sanger report for three of the five discordant bases, one was shown to be a false positive supporting the WES data, and one result differed from both the Sanger and WES data. Two of the discordant zygosity results supported Sanger and the other two supported WES data. We report high concordance for well‐covered coding variants, supporting the use of WES as a screening tool for heterogeneous disorders, and recommend the use of supplementary Sanger sequencing for poorly‐covered genes when the clinical suspicion is high. Importantly, despite remaining difficulties with achieving complete coverage of the whole exome, 10 (38.5%) of the 26 compared patients were diagnosed through WES.

## Introduction

Although rare diseases are by definition individually uncommon, they collectively affect one in 50 individuals (Orphanet 2014). Of the approximately 7000 rare genetic diseases, the molecular etiology of over 4500 have now been identified (OMIM, www.omim.org; accessed 19 December 2015), enabling the diagnosis of patients with these often devastating disorders. A definitive molecular diagnosis facilitates informed prognosis, disease management, recurrence risk counseling, and genetic testing of at‐risk family members. For the past two decades the “gold standard” for clinical DNA sequencing has been the automated Sanger method. Although Sanger sequencing is considered the most reliable method of sequencing, it limits genetic testing to a single or few genes at a time; becoming costly and time‐consuming when multiple genes are tested before reaching a diagnosis. Given the steady increase in the number of recognized disease genes (Boycott et al. 2013), many with similar or overlapping clinical presentations, the sequencing of individual genes is becoming less and less practical for disorders with no single strong candidate gene to interrogate.

Whole‐exome sequencing (WES) is a next‐generation sequencing (NGS) strategy that isolates the majority of the protein‐coding portion of the genome and is emerging as a diagnostic tool for patients with undiagnosed rare diseases. Although coding regions comprise only a small portion of the entire human genome, mutations in these regions are estimated to account for 85% of monogenic diseases (Dixon‐Salazar et al. 2012). The ability to analyze all genes simultaneously makes WES an effective method for both novel disease gene discovery and the efficient diagnosis of known genetic diseases, with reported diagnostic rates approaching 30% (Yang et al. 2013, 2014; Iglesias et al. 2014; Lee et al. 2014; Farwell et al. 2015). WES is particularly useful for the diagnosis of conditions associated with significant genetic heterogeneity, where Sanger methods can become cumbersome and costly with concomitant lower rates of diagnosis (Ku et al. 2012; Sawyer et al. 2014). A retrospective study by Neveling et al. (2013) demonstrated that WES had a consistently higher diagnostic yield than routine clinical Sanger sequencing for five genetically heterogeneous disorders; and concluded that when patients require more than three Sanger‐based tests to achieve a diagnosis, WES becomes a more cost‐effective method.

As with any NGS method, WES still has some limitations leading to poor coverage or sequencing inaccuracy. Poor targeting by the exome capture kit, high guanine‐cytosine (GC) content, and the presence of repetitive sequences can all affect WES coverage and sequencing alignment. Inaccuracy in WES base‐calling can also be caused by allelic dropout – the failure to amplify one or both alleles at a specific locus.

While a number of studies have reported on the validity of targeted NGS panels designed for the assessment of genes related to a particular genetically heterogeneous disease (e.g., cardiomyopathy), very few have directly compared WES to clinical Sanger sequencing. For example, Baudhuin et al. (2015) evaluated the concordance of four targeted NGS panels, for hereditary colon cancer, arrhythmias, cardiomyopathies, and other cardiovascular‐related genes, to identify 919 variants identified in 117 genes by Sanger sequencing and demonstrated 100% concordance. This result is perhaps not surprising, given that the panels studied were specifically designed to have complete coverage of a limited number of genes. The comparison of 137 pathogenic variants identified by Sanger sequencing to WES results for neuromuscular disease genes determined that up to 18% of pathogenic variants in these genes were poorly covered by WES, but did not report on the specific concordance of base‐calling (Ankala et al. 2015). Further comparison of WES to Sanger sequencing would provide useful information for clinicians when deciding between these two methods of patient testing.

Two national research programs in Canada, Finding of Rare Genetic Disease Genes (FORGE; Beaulieu et al. 2014), and its successor, Enhanced Care for Rare Genetic Diseases in Canada (Care4Rare), use WES to provide definitive molecular diagnoses to both pediatric and adult patients with rare diseases. All research findings are reported back to the referring clinicians and confirmed in clinically certified molecular diagnostic laboratories. Using WES data from participants in the FORGE and Care4Rare projects, we retrospectively examined the concordance of WES and Sanger sequencing data by performing a direct comparison of 260 variants identified by clinical Sanger sequencing to the corresponding WES results in 26 patients when they entered one of these projects. Using the reported variants of previous clinical Sanger sequencing in these patients, polymorphic variants and variants of unknown significance were compared to the corresponding WES results to observe if they had been identified by WES. Discordant results were further investigated using Sanger sequencing to determine whether the original clinical Sanger sequencing test or WES had made the correct call. Using this data, we were able to estimate the concordance rate of these two methods.

## Methods

### Participant selection

All study participants were identified by their primary clinician at the Children's Hospital of Eastern Ontario (CHEO) and had previously undergone WES as part of either FORGE of Care4Rare; both studies were approved by the CHEO Research Ethics Boards and informed consent was obtained from all families.

In this retrospective study, the charts of all potential participants were reviewed to determine if prior clinical Sanger sequencing had been performed for one or several genes. Clinical molecular reports were queried for specific single nucleotide variants (SNVs), small insertions, and small deletions reported by Sanger sequencing. Patients with no reported variants were excluded.

### Chart review

Of the 109 potential participants identified, 88 had clinical charts readily available for review of test results, and of those 26 had molecular testing by Sanger sequencing with reported variants that met criteria for inclusion in the study. All variants reported by clinical Sanger sequencing were recorded; including the test performed, diagnostic laboratory, gene name, nucleotide and chromosomal positions, amino acid change, and reference identification for the NCBI single nucleotide polymorphism database (dbSNP; Sherry et al. 2001), where applicable. Chromosomal positions that were not reported in diagnostic reports were determined using the Leiden University Medical Centre Mutalyzer program (Wildeman et al. 2008).

### Sequencing

Clinical Sanger sequencing was performed in different accredited facilities within Ontario and internationally. Sanger sequencing evaluation of discordant variants was completed at the CHEO Research Institute in Ottawa, Canada. WES through the FORGE and Care4Rare projects was performed at the McGill University and Genome Quebec Innovation Centre (Montreal). Targeted exon capture was performed using the Agilent SureSelect All Exon 50 MB (V3 or V4) exome enrichment kit. Captured fragments were sequenced by the Illumina HiSeq 2000 platform in 100 bp paired‐end reads, producing a minimum of 10 Gb of sequence for each sample. An average coverage of 80× for each sample was required for the data to pass quality control and be analyzed. Short sequence reads were preprocessed using the FASTX Toolkit Quality Trimmers (http://hannonlab.cshl.edu/fastx_toolkit/). Sequence reads were aligned to hg19 using BWA 0.5.9 (Li and Durbin 2010), insertion and deletion realignment was performed by the Genome Analysis Toolkit (GATK; McKenna et al. 2010), and duplicate reads identified and excluded using Picard (http://picard.sourceforge.net/). Our algorithm requires a minimum of three reads in order for a nucleotide position to be called. Coverage of consensus coding sequence (CCDS) was evaluated using GATK, which typically showed that our samples have over 94% of CCDS bases with coverage over 10x, and over 90% of CCDS bases with coverage over 20×. For each sample, SNVs, short insertions, and short deletions were identified by SAMtools MPileup (Li et al. 2009) with the extended base alignment quality adjustment, and requiring a minimum of 20% of sequencing reads supporting the base call. Variant calling was made using the Human Genome Variation Society mutation nomenclature.

### Variant comparison

Variants identified by clinical Sanger sequencing were manually compared to the corresponding unfiltered Variant Call Format (VCF) file containing all variants identified by WES. The corresponding variants in the VCF files were confirmed as correct using chromosomal position, nucleotide position, gene name, and reference identification from the clinical report. Variants with a read depth of 10× or less were noted. For the variants that were missing in WES results, the coverage of the specific chromosomal position was analyzed using WES Binary Alignment/Map (BAM) files. Variants in poorly‐covered genes, defined here as having less than 75% of sequence with coverage of greater than 20×, were excluded from further analysis.

## Results

After reviewing the clinical charts of 109 FORGE and Care4Rare participants, we identified 26 patients who had clinical Sanger sequencing in which one or more SNVs, small insertions, or small deletions were reported. In total, we identified 391 variants (coding [208] [Table 1] and noncoding [183]) that could be used to evaluate the concordance to WES. Nine genes were found to be poorly covered (defined as less than 75% of the gene with coverage greater than 20×) in WES data from 15 different patients: *CCD2D2A*,* DES*,* DOK7*,* EPM2A*,* FKRP*,* KCNC3*,* LMNA*, and *RAPSN*. Within these low‐coverage genes, 79 of 131 variants were identified in the WES data, resulting in a concordance rate of 60.3%. As lack of coverage is a well‐known limitation of WES that is constantly being improved by new exome capture kits, variants in these consistently poorly‐covered genes were excluded from further analysis. Of the remaining 260 variants (150 coding and 110 noncoding) in 42 different genes, 236 (90.8%) were confirmed in WES results. Of the 150 coding variants, 146 (97.3%) were seen in WES results; whereas only 90 of 110 noncoding variants (81.8%) were present in WES (Table 2). The concordance of clinical Sanger sequencing to WES variants was lower in intronic regions within 20 bp of the nearest exon (92.3%); and lowest in deeper intronic regions (75.9%). The latter results are expected, as only the coding portion of the genome is targeted by WES. Few concordant variants had low coverage; only three coding and six intronic variants were covered by fewer than 10 reads.

**Table 1:** Concordance between Sanger and WES coding variants in individual genes

Genes	Sanger	WES
GAA	35	9
DYSF	13	13
POMT2, GALC	10	10
ZFYVE26	9	9
FKTN, POMT1, TTN	8	8
DES	7	7
KCNA1, SLC12A6	6	6
COX10, KIAA1840, SGCG, CHAT, CACNA1A	5	5
RAPSN	5	4
CAPN3, SGCD, TCAP	4	4
CLN10, DOK7	3	3
MUSK	3	1
FKRP, LMNA, CC2D2A, CLN2, EIF2B5, FAM134B, KCNC3, POMGnT1, MYH7	2	2
APTX, ARL13B, CHRND, CHRNE, CLN1, CLN5, COL6A2, EIF2B4, EPM2A, NPHP1, PLEKHG4, SACS, SCO2, SETX, SPG7, SYNE1, TRPV4	1	1
COLQ	1	0
TMEM67	1	0
Total	208	177

Sanger indicates the number of variants identified by clinical Sanger sequencing, and WES indicates the number of variants confirmed by whole‐exome sequencing.

**Table 2:** Clinical Sanger variants compared to whole‐exome sequencing (WES) variants in 42 adequately covered genes

Variants	Total variants	Concordant variants (%)	Discordant variants (%)	Number of patients
Coding	150	146 (97.3%)	4^b^ (2.7%)	21
Intronic (≤20 bp)	52	48 (92.3%)	4 (7.7%)	13
Intronic (>20 bp)	58^a^	44 (75.9%)	13 (22.4%)	14

Genes with less than 75% coverage of more than 20× were excluded, eliminating *CCD2D2A*,* DES*,* DOK7*,* EPM2A*,* FKRP*,* GAA*,* KCNC3*,* LMNA*, and *RAPSN* from comparison.

a One intronic variant was miscalled by both Sanger and WES by repeat Sanger sequencing; therefore, is neither concordant nor discordant.

b One discordant coding variant was shown to be a false positive by repeat Sanger sequencing.

**Figure 1:**
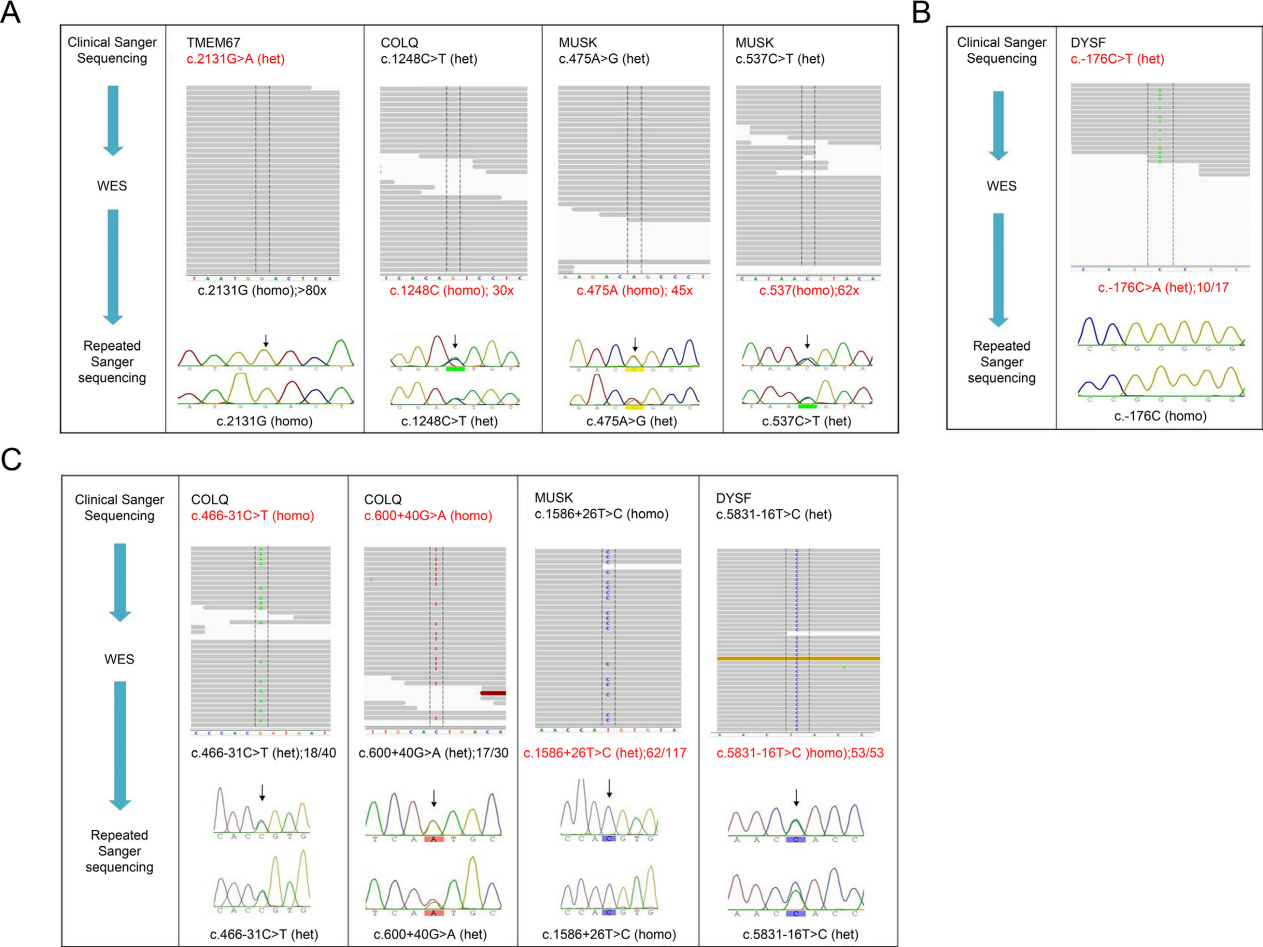
Discordant whole‐exome sequencing (WES) variants from 42 adequately covered genes. Representation of WES coverage of each variant position viewed in Binary Alignment/Map files through IGV, outlined by dashed lines (upper). Variant calls are labeled on individual sequence reads; colored bands indicate lower quality reads. Electropherograms display the repeat Sanger sequencing, with the variant positions indicated by black arrows (lower). *Het* indicates that the variant is heterozygous in the patient, while *homo* represents a homozygous variant. A) Discordant coding bases (GenBank Accession Numbers: *TMEM67* NM_153704.3; *COLQ* NM_005677.3; *MUSK* NM_005592.3). B) Discordant noncoding base (GenBank Accession Numbers: *DYSF* NM_001130978.1). C) Discordant zygosities (GenBank Accession Numbers: *COLQ* NM_005677.3; *MUSK* NM_005592.3; *DYSF* NM_001130978.1).

The majority of the 150 coding variants were SNVs; three were small deletions, ranging from one to five nucleotides in size. Of the 52 intronic variants, less than 20 bp from the nearest exon, there were three small deletions and one small insertion. The intronic variants further than 20 bp from the nearest exon included five small deletions and two small insertions. All of the insertions and deletions identified by clinical Sanger sequencing were concordant with WES results. Four of the coding SNVs identified by clinical Sanger sequencing in the 42 adequately covered genes were not present in WES results. These four discordant variants were seen in three genes (*TMEM67*,* COLQ*, and *MUSK*), and isolated to two patients (Fig. 1). Repeated Sanger sequencing confirmed the presence of three of the four SNVs, however, the variant identified by clinical Sanger sequencing in *TMEM67* was shown to be a false positive, supporting the WES result. Further analysis of the WES data showed the positions of the three missing variants to be fairly well‐covered, with read depths of 30 to 60×, having consistently called the sequence as matching the reference sequence rather than the variants identified by Sanger sequencing (Fig. 1A).

One other discordant base was identified in the 5′ untranslated region of the gene *DYSF* (Fig. 1B). This base was called differently by clinical Sanger sequencing and WES, but was shown to be wild‐type by our confirmatory Sanger sequencing.

In addition to the discordant coding variants, there were four variants identified by Sanger sequencing that were present in the WES results, but the WES and Sanger sequencing data were discordant as to whether the variants were homozygous or heterozygous (Fig. 1C). In two cases, repeat Sanger sequencing confirmed the clinical Sanger results – one of which was homozygous and the other heterozygous. However, repeat Sanger sequencing supported the WES results of two other variants; both of which were heterozygous.

## Discussion

One of the most significant challenges of WES as an emerging diagnostic tool is achieving sufficient coverage of disease‐relevant genes; as demonstrated in our results, nine of 51 disease genes analyzed (17.6%) were found to be poorly covered by WES when evaluated from the perspective of concordance with clinical Sanger data. Previous studies have found that 5‐10% of genes sequenced in WES may be poorly covered and considered to be low‐quality sequences (Neveling et al. 2013; Tétreault et al. 2015). Regions of sequence with high GC content can be more difficult to capture, with some capture kits providing better coverage of these regions than others (Hoischen et al. 2010; Ku et al. 2012). GC content of these nine genes was calculated using Ensembl Genome Browser sequences and EndMemo DNA/RNA GC Content Calculator (http://www.endmemo.com/bio/gc.php). While average GC content is around 37%, seven of the poorly‐covered genes had GC content greater than 50% (*DOK7*,* KCNC3*,* GAA*,* DES*,* RAPSN*,* LMNA*, and *FKRP*), which may explain their low coverage in WES (Hoischen et al. 2010; Cunningham et al. 2015). The remaining genes, *CC2D2A* and *EPM2A* had GC content closer to 40%, suggesting that their low coverage may be an artifact of poor targeting by the exome capture kits used in this study.

In the 42 genes with adequate coverage (over 75% of sequence with coverage of greater than 20×), four of the 150 assessed coding variants were discordant; however repeat Sanger sequencing showed one of the variants to be a false positive in clinical Sanger sequencing (Fig. 1A). The three confirmed discordant coding variants were observed in only one patient, corresponding to one Sanger sequencing panel; and all three variants were heterozygous. It is possible that WES may have selectively amplified the reference allele over that of the variant allele, causing the WES analysis and annotation to call it as wild‐type. Interestingly, all three discordant variants were SNVs rather than insertions or deletions; the latter are generally considered to be more difficult to sequence using NGS methods.

An additional variant, in Patient 3, was called as heterozygous by both clinical Sanger sequencing and WES, but one identified the altered nucleotide as a thymine and the other as an adenine (Fig. 1B). Interestingly, repeat Sanger sequencing determined that there was no variation of sequence, but that the patient was homozygous for the reference nucleotide. This variant was located 176 nucleotides upstream of the gene; therefore, would be expected to be poorly captured in WES and may not have been a relevant target sequence in the original Sanger sequencing either.

Detailed analysis of WES coverage revealed that the positions of the missed variant calls were generally well‐covered in WES, with coverage of greater than 30 reads each. Therefore, among adequately covered genes, we have demonstrated 97.3% concordance between WES and clinical Sanger sequencing in identifying SNVs, small insertions, and small deletions. Because one of the 150 coding variants (0.7%) is a false positive in clinical Sanger sequencing, this equates to a false negative rate of 2.0% (3 of 150) for WES; with the caveat that we are not using the traditional definition of false positive and false negative, which typically refers to clinically relevant results (either falsely identified or missed) and not polymorphisms or variants of unknown significance.

In four instances, the clinical Sanger sequencing and WES results were discordant with respect to the homozygous or heterozygous nature of the variant (Fig. 1C). Repeat Sanger sequencing confirmed the clinical Sanger results in two of the variants – one of which was homozygous and the other heterozygous. However, Sanger sequencing supported the WES results of two other variants; both of which were heterozygous. Therefore, the homozygous calling of these two variants by Sanger sequencing could represent primer bias in the clinical test, where one allele was predominantly amplified over the other during PCR amplification. Notably, three of the discordant coding variants and three of the homozygous/heterozygous discrepancies occurred in Patient 5, and both included the *COLQ* and *MUSK* genes. Two of the *COLQ* variants called by WES were supported by repeat Sanger sequencing; however, there was also a large portion of the gene missing in the WES data, likely indicative of poor‐quality DNA in this sample. In addition to incorrect calling of variants, poor DNA quality may also result in lower overall coverage and shifts in allelic ratios (Guo et al. 2014). It should also be noted as a potential limitation of this study that there may be an inherent bias in validating discordant variants by repeating Sanger sequencing, as the possible discrepancies of the clinical Sanger sequencing could be inadvertently repeated.

Our study assessed variants identified by clinical Sanger sequencing as concordant or discordant in WES data from the same patient. Given that all patients were subsequently studied in the FORGE or Care4Rare projects, and were thus undiagnosed, the variants compared in this study are mainly common polymorphisms observed in the general population, but were reported as part of their clinical Sanger sequencing reports. Of the 26 patients analyzed, 10 (38.5%) were diagnosed through WES – eight patients were found to have mutations in known disease‐causing genes and two patients were solved by the identification of novel disease genes (Table 3). Each of the novel disease genes were discovered using a different strategy. The discovery of *DDHD2* was supported using genetic validation with the identification of three other affected families with similar features of complex hereditary spastic paraplegia (Schuurs‐Hoeijmakers et al. 2012). In contrast, the discovery of *LIMS2* was based on a single family exhibiting limb‐girdle muscular dystrophy and required model system correlation to support disease gene pathogenicity (Chardon et al. 2015). One additional patient may be potentially explained by a novel gene, requiring further evidence, while three patients are currently in the data analysis pipeline. The final 12 patients remain unsolved after initial WES analysis, with multiple potential candidates requiring further investigation.

**Table 3:** Clinical testing and whole‐exome sequencing (WES) outcome of patient cohort

Disorder	Sanger sequencing (individual genes)	Sanger sequencing (gene panels)	MLPA and expansion testing	Status	WES diagnosis
Early‐onset generalized dystonia	PRKRA, THAP1, DYT1, GCH1, MERRF, APTX		SCA, DRPLA	Solved (known gene)	ATM
Neonatal epileptic encephalopathy	CSTB	Myoclonus epilepsy panel (4), Neuronal ceroid lipofuscinosis panel (8)	CSTB, DRPLA	Solved (known gene)	ASAH1^b^
Nuclear encoded mitochondrial disorder	TAZ, SCN5A	Dilated cardiomyopathy panel (27)		Solved (known gene)	GARS^c^
Muscular dystrophy NYD	COL6A1	Congenital muscular dystrophies panel (3), Limb‐girdle muscular dystrophy panel (9)		Solved (known gene)	COL6A1^a^
Emery–Dreyfuss phenotype	EMD, LMNA			Solved (known gene)	COL6A1
Hennekam‐like syndrome	CCBE1			Solved (known gene)	KMT2A
Cerebellar ataxia	SYNE1, SACS			Solved (known gene)	CACNA1A
Mitochondrial disorder		Austosomal recessive ataxia panel (6)		Solved (known gene)	ITPR1
Limb‐girdle muscular dystrophy with triangular tongues		Limb‐girdle muscular dystrophy panel (10)		Solved (novel gene)	LIMS2^d^
Hereditary leg dominant quadriparesis	MCOLN1, KIAA1840, ZFYVE26			Solved (novel gene)	DDHD2^e^
Developmental delay and hereditary spastic paraplegia	APTX, EIF2B1/2/3/4/5, GALC, GJC2	Hereditary spastic paraplegia panel (10), Neuronal ceroid lipofuscinosis panel (8)		Candidate (novel gene)	SYNJ2
Muscular dystrophy, congenital, with cerebellar atrophy	KCNC3, SACS, APTX, SETX, POLG1, SIL1, TTPA		SCA, DRPLA, FXN, FMR1	Analysis	–
Coloboma‐ectodermal hypotonia	PORCN	Congenital muscular dystrophies panel (4)		Analysis	–
Ataxia	PLEKHG4, SPTBN2			Analysis	–
Hereditary spastic paraplegia, intellectual disability, thin corpus callosum	ZFYVE26, KIAA1840, PANK2	Hereditary spastic paraplegia panel (6)		Unsolved	–
Fitzsimmons–Guilbert syndrome	SACS			Unsolved	–
Alternating hemiplegia	ATP1A3, ATP1A2, CACNA1A, KCNA1, CACNB4, SLC1A3			Unsolved	–
Distal myopathy	FHL1, CRYAB, DES, MYH7, GNE, FSHD	Limb‐girdle muscular dystrophy panel (13)		Unsolved	–
Joubert syndrome		Joubert/Meckel–Gruber syndrome panel (8)		Unsolved	–
Hereditary sensory autonomic neuropathy with developmental delay	FAM134B, HSN2			Unsolved	–
Congenital myasthenia		Congenital myasthenic syndrome panel (9)	OPMD	Unsolved	–
Rapidly progressive myopathic disorder	DMD, COL6A2, GAA, SEPN1, TK2	Congenital myasthenic syndrome panel (5), Mitochondrial panel, Limb‐girdle muscular dystrophy panel (9)	FHSD	Unsolved	–
Mitochondrial disorder	DGUOK, SCO2, SURF1, UGT1A1, FASTKD2, COX10, COX6B1, COX15, SCO1			Unsolved	–
Basal ganglia strokes		Neuronal ceroid lipofuscinosis panel (8)		Unsolved	–
Charcot–Marie‐Tooth disease	TRPV4			Unsolved	–
Charcot–Marie‐Tooth disease	HMBS, AIFM1, GJB1, SLC12A6		SMN1	Unsolved	–

a WES identified a splicing defect that was missed by clinical immunolabeling of muscle biopsy.

b Dyment et al. 2014

c McMillan et al. 2014

d Chardon et al. 2015

e Schuurs‐Hoeijmakers et al. 2012

Although the complete coverage of the exome remains a challenge, the continuing improvement of exome capture kits should facilitate more consistent coverage in the future. Improvement to capture kits would include targeting of areas of consistently low coverage, and the optimizing of capture to prevent strand bias. Increased coverage has already been observed in more recent capture kits, with adequate coverage of almost 95% of the exome reported using the Agilent V5 kit (Lelieveld et al. 2015); the development of new capture kits will only further improve the coverage. Therefore, a notable limitation to our retrospective study is the use of Agilent V3 and V4 capture kits in all WES samples. Despite significant progress toward optimizing WES‐based approaches for clinical care, it remains difficult to achieve complete coverage of the entire exome. Therefore, WES should be considered a useful screening tool for genetically heterogeneous disorders, and clinical diagnostic laboratories should report genes that are consistently not adequately covered, as well as those not adequately covered for a particular patient sample relevant to the clinical indication for testing; additional Sanger sequencing of individual genes or gene panels should be used to supplement any poorly‐covered genes where the clinical suspicion is high that the gene is causative.

In conclusion, we have demonstrated that where coverage is sufficient, WES has high concordance with Sanger sequencing; as shown by our analysis of 42 adequately covered genes in which 97.3% of variants were concordant. Occasionally, as was the case with three heterozygous variants, WES may be more accurate than Sanger sequencing. Given the increasing affordability and efficiency of sequencing the entire exome at once, WES should be considered as an alternative to Sanger sequencing for patients with genetically heterogeneous disorders, where the sequencing of individual genes becomes slow and costly. However, our findings also highlight that when there is strong suspicion of particular genes based on clinical presentation, the coverage attained by WES of those specific genes should be scrutinized and the information included in the patient report. Laboratories reporting WES results should also establish standards for reporting such poorly‐covered genes. Relevant genes that are found to be poorly covered in WES may be resequenced by targeted NGS or Sanger sequencing, to reduce the incidence of clinically relevant false negatives. For patients with a specific phenotype associated with significant genetic heterogeneity (for example, Charcot–Marie‐Tooth disease), clinicians might also consider the use of targeted NGS panels that guarantee coverage of the clinically relevant genes. The trade‐off in this is of course the inability of such panels to be reanalyzed for recently reported disease genes associated with the particular primary indication. With clinical laboratory quality measures in place, and clinical capture kits as well as standardized analysis pipelines constantly improving, WES will enable affordable and efficient diagnosis of rare genetic diseases.

## Conflict of interest

None declared.
